# Progress in pediatrics in 2013: choices in allergology, endocrinology, gastroenterology, hypertension, infectious diseases, neonatology, neurology, nutrition and respiratory tract illnesses

**DOI:** 10.1186/1824-7288-40-62

**Published:** 2014-07-12

**Authors:** Carlo Caffarelli, Francesca Santamaria, Alessandra Vottero, Carlotta Povesi Dascola, Virginia Mirra, Francesco Sperli, Sergio Bernasconi

**Affiliations:** 1 Department of Clinical and Experimental Medicine, Clinica Pediatrica, University of Parma, Parma, Italy; 2 Department of Pediatrics, Federico II University, Naples, Italy

**Keywords:** Allergology, Endocrinology, Gastroenterology, Hypertension, Infectious diseases, Neonatology, Neurology, Nutrition and respiratory tract illnesses in children

## Abstract

This review will provide new information related to pathophysiology and management of specific diseases that have been addressed by selected articles published in the *Italian Journal of Pediatrics* in 2013, focusing on allergology, endocrinology, gastroenterology, hypertension, infectious diseases, neonatology, neurology, nutrition and respiratory tract illnesses in children. Recommendations for interpretation of skin prick test to foods in atopic eczema, management of allergic conjunctivitis, hypertension and breastfeeding in women treated with antiepileptic drugs and healthy breakfast have been reported. Epidemiological studies have given emphasis to high incidence of autoimmune disorders in patients with Turner syndrome, increasing prevalence of celiac disease, frequency of hypertension in adolescents, incidence and risk factor for retinopathy of prematurity. Advances in prevention include elucidation of the role of probiotics in reducing occurrence of allergies and feeding intolerance, and events of foetal life that influence later onset of diseases. Mechanistic studies suggested a role for vitamin D deficiency in asthma and type 1 diabetes and for reactivation of Varicella-Zoster virus in aseptic meningitis. Regarding diagnosis, a new mean for the diagnosis of hyperbilirubinaemia in newborns, a score for recognition of impaired nutritional status and growth and criteria for early Dyke-Davidoff-Masson Syndrome have been suggested. New therapeutic approaches consist of use of etanercept for reducing insulin dose in type 1 diabetes, probiotics in atopic eczema, and melatonin in viral infections.

## Introduction

In the last year, numerous exciting advances in pediatrics have been reported in the *Italian Journal of Pediatrics*. This review will focus on 2013 publications aiming to underline the last updates in the fields of allergology, endocrinology, gastroenterology, hypertension, infectious diseases, neonatology, neurology, nutrition and respiratory tract illnesses in children.

### Allergy and respiratory tract illnesses

The major focus of last year journal was on food allergy, allergic conjunctivitis and asthma. Probiotics have been used for numberless diseases. Castellazzi AM et al. [1] provided a valuable review of the effects of probiotics in preventing and/or treating allergic disorders, discussing the contradictory results of 14 studies and 1 meta-analysis on probiotics efficacy in allergy prevention. Moreover, promising findings of 6 studies on the treatment of atopic eczema with probiotics were reported. Further investigations are needed to clarify strains, duration and doses of treatment that should allow reaching the best clinical results.

In infants with atopic eczema, skin prick test results are often positive. Their practical usefulness is a controversial issue that was reviewed by Caffarelli C et al. [2]. Skin tests may be helpful for suspicious foods to which eczematous patients will react. However, reactions after exposure to foods to which IgE tests are positive occur in 30–60% of children with atopic eczema, mostly when atopic eczema is moderate-to-severe. Therefore, the efficacy of exclusion diet led by positive SPT to foods must be confirmed by oral food challenge. The challenge is also warranted to avoid unnecessary diet restrictions that may favor the loss of immunologic tolerance and the onset of food allergy. This was observed in children affected by atopic eczema. After a long period of exclusion diet, they had anaphylactic reactions to cow’s milk that had never occurred before the diet. Finally, in eczematous infants, positive skin prick test results to hen’s egg or peanuts that were not previously ingested are useful to predict the risk of immediate reactions at the first known intake.

Classification and management of allergic conjunctivitis was comprehensively summarized by La Rosa M et al. [3]. A useful synoptic view of the possible mechanisms and clinical features was provided. They highlighted the role of anti-H1 anti-histamines, decongestants and mast cell stabilizers as first option treatment in seasonal and perennial allergic conjunctivitis. Non-steroidal anti-inflammatory agents, corticosteroids and immunotherapy are preferable as second line treatment. Some markers of allergy may be found in vernal keratoconjunctivitis and in atopic keratocongiuntivitis that are less common, but potentially more severe. Allergists are mainly consulted by those patients, but the involvement of pediatric ophthalmologists may be necessary to avoid preventable vision loss in severe cases. Most people are aware from media reports that vitamin D deficiency can predispose to the occurrence of atopic diseases. Uysalol M et al. [4] reported that asthmatic children had significant lower vitamin D levels than healthy children in Tekirdag, Turkey. Decreased vitamin D levels were associated with difficult-to-control asthma, increased number of emergency unit admissions, number of hospitalizations, severity of asthma. Of interest, asthmatic children had less exposure to sunlight and ate a diet less rich in vitamin D. This may indicate that asthma control may benefit from a healthier life-style and a vitamin D supplementation. However, if we want to give such recommendations, we have to provide solid evidence.

### Endocrinology

There has been considerable interest in diagnostic and therapeutic challenges presented by patients with autoimmune endocrine diseases. Recent studies suggest that vitamin D deficiency may increase the risk of developing autoimmune diseases including type 1 diabetes (T1DM), by modulating the immune and inflammatory reactions. In particular, at the level of the immune system, 1, 25(OH)2 D3 prevents the transformation of dendritic cells into antigen presenting cells, and at the level of the pancreatic islets, it decreases proinflammatory chemokine and cytokine expression (e.g., IL6) resulting in decreased T cell recruitment and infiltration, increased regulatory cells, and arrest of the autoimmune process. Azab SFA et al. [5] assessed vitamin D status in Egyptian children and adolescents with T1DM in order to clarify the prevalence of vitamin D deficiency and its role in the pathogenesis of T1DM. Although the small sample size (80 T1DM cases aged 6 to 16 years and 40 healthy children age and gender matched as control group), the authors found that serum vitamin D levels were not significantly lower in diabetic subjects compared to the control group (24.7 ± 5.6 *vs* 26.5 ± 4.8 ng/ml; P > 0.05). Among diabetic cases, 55% were vitamin D deficient, while 45% had normal vitamin D level (P < 0.01), leading to a significant difference as regards of HOMA-IR and diabetes duration (P < 0.01). The authors conclude that public health message on the importance of vitamin D status especially in diabetic children and adolescents, should be disseminated to the public.

Olivieri AN et al. [6], describe the case of a girl suffering from Type 1 diabetes mellitus (T1DM) and Autoimmune Hashimoto's Thyroiditis (AHT) since the childhood. Due to the onset of Juvenile Idiopathic Arthritis (JIA) during adolescence the patient was treated for three years with an anti-Tumor Necrosis Factor-alpha drug, etanercept, and the authors observed a significative reduction of insulin dose although a well-controlled diabetes, normalization of her inflammatory markers, and inactivation of the arthritis. This case report demonstrates that etanercept is really effective and safe for the treatment of JIA associated with T1DM and AHT. However, further studies of longer duration on a larger population are needed to assess the role of biologic drugs and immunotherapy in this group of patients.

An increased frequency of autoimmunity as well as an elevated incidence of autoantibodies has been observed in patients with Turner syndrome. Grossi A et al. [7] retrospectively showed that out of the 66 Turner patients, 26 had thyroid autoimmune disorders (39.4%), 14 patients had Hashimoto’s thyroiditis with clinical or subclinical hypothyroidism (21.2%) and 12 patients had circulating anti-thyroid antibodies, echographic pattern of diffuse hypoechogenicity and normal thyroid hormone levels (18.2%). None were affected by Graves’ disease. Since thyroid autoimmunity was the most frequent autoimmune disorder, the authors analyzed the overall incidence of thyroid autoimmunity within the 3 different age groups 0–9.9, 10–19.9 and 20–29.9 years, but no statistically significant difference was observed. When comparing the incidence of thyroid autoimmunity among the various karyotype groups (45, X; mosaicism/deletion/ring chromosome; X isochromosome), a statistically significant difference was observed, being higher in the X isochromosome group compared to the others. These data confirm a high frequency of thyroid autoimmunity in Turner patients, suggesting as an early assay of autoantibodies and monitoring thyroid hormones is fundamental for detecting hypothyroidism earlier and start adequate replacement therapy.

### Gastroenterology

In last year journal, attention was paid to prevention of celiac disease and feeding intolerance in neonates. In the Republic of San Marino, a population-based screening programme for celiac disease was performed mainly in 6 years old children [8]. It was found a prevalence of celiac disease that was comparable to other European countries but, interestingly, it showed an increase from 0.7% before 2005 to 1.8% after 2005. Concordance between anti-transglutaminase IgA antibodies (ATTG) and anti-endomysial antibodies (EMA) was near 100%. The cost of screening was 20 euro per patient. However, the benefits for the population were not easy to calculate, and this would be a pity since this screening would represent an important opportunity to intervene early in the course of the disease. There is a lot of interest in the role of microbial biodiversity of infant gut between breast-fed infants, bottle-fed infants, preterm and infants born via caesarian section in predisposing to diseases.

A review by Di Mauro A et al. [9] on this topic underlined that the intestinal *microbiota* might help not only in the maintenance of barrier function inducing an increased epithelial cell proliferation and integrity, and the development of the intestinal villus, but also in both sensory and motor gut functions, and regulation of visceral pain depending from the gut-brain axis. So, gut microbiota influences post-natal gastrointestinal functions such as nonspecific defensive mechanisms, innate and acquired immune response, development of GALT that secretes immunoglobulin A (IgA) and modulates oral tolerance i.e. to food allergens. There are several potential negative outcomes that can be explained by an abnormal early colonization. It may induce an unbalanced T cell (Th2 > Th1 or Th1 > Th2) response resulting in feeding intolerance in early postnatal life and in gastrointestinal disease in childhood. It may be involved in lack of distinction between dangerous and harmless antigens, mucosal inflammation, and altered intestinal permeability. In premature infants, the abnormal colonization may favor feeding intolerance. There is therefore a need of studies on benefits of early probiotic supplementation.

### Infectious diseases

Melatonin is a versatile molecule which is synthesized by the pineal gland. Besides playing an important role in various functions of the body, including sleep and circadian rhythm regulation, melatonin has been found to have also important immunomodulatory and antioxidant properties. Because of these latter characteristics, Silvestri M et al. [10] showed that melatonin has also been found to be effective in fighting viral infections in a variety of experimental animal and in vitro studies, suggesting a potential novel pharmacological approach to ameliorate the host reactions against viral infections and their long-term consequences. The use of pharmacological concentrations is essentially non-toxic in humans, also in the neonatal period, although one possible weakness of melatonin is its short half-life and the relatively low levels in serum during day hours.

Neurological complications (aseptic meningitis, myelopathy, vasculopathy, eye disease) of varicella zoster virus (VZV) reactivation are relatively uncommon in children, most of the cases being described in the adults or elderly patients population. Esposito S et al. [11] described an immunocompetent 14-years-old adolescent with aseptic meningitis due to VZV reactivation that rapidly improved after intravenous acyclovir administered for 10 days. A brain magnetic resonance imaging performed immediately after the discontinuation of the treatment and a clinical examination carried out approximately four weeks later showed no signs or symptoms of active disease. Aseptic meningitis is one of the possible complications of VZV reactivation, and can also affect immunocompetent children. In the current case, although the neurological involvement was clinically evident, it was indeed very mild and did not cause any persistent damage, possibly because oral antiviral therapy was promptly started as herpes zoster was diagnosed.

### Hypertension

Hypertension is the prevalent cardiovascular disease risk factor worldwide. Therefore, recommendations on this issue provided by Spagnolo A et al. [12] on behalf of the Italian Society of Pediatrics and the Italian Society of Hypertension were quite appreciated. The document was based on previous rules published in 2009 by the European Society of Hypertension and it was adapted to the Italian health care system. It focused mainly on primary hypertension which represents a growing problem in children and adolescents. Subjects at elevated risk of hypertension are those overweight, with low birth weight and presenting a family history of hypertension. However, also children who do not present these risk factors may have elevated blood pressure levels. The prevention of high blood pressure is based on correct lifestyle and nutrition, starting from childhood age. The treatment of primary hypertension in children is almost exclusively dietary/behavioral and includes: i) reduction of overweight whenever present; ii) reduction of dietary sodium intake; iii) increase in physical activity. Pharmacological therapy is needed rarely and only in specific cases.

Although hypertension was believed to be mainly diagnosed among middle aged and elderly patients, this study conducted by Ujunwa A et al. [13] showed that a relatively high prevalence rate of hypertension and prehypertension was present among secondary school adolescents in Enugu South East Nigeria (2694 adolescents aged 10–18 years). In particular, the mean systolic blood pressure and diastolic blood pressure were 106.66 ± 11.80 mmHg and 70.25 ± 7.34 mmHg, respectively, in males, and 109.83 ± 11.66 mmHg and 72.23 ± 8.26 mmHg, respectively, in females (P < 0.01). Blood pressure differed between males and females, and in fact, the prevalence of hypertension and prehypertension was higher in females (6.9% and 20.1%, respectively) than males (3.8% and 14.3%, respectively). Blood pressure increased with age with a prevalence of obesity of 1.9%. The authors concluded that periodic screening and monitoring of blood pressure of adolescents should be done, and since modifiable risk factors exist among adolescents, early lifestyle modification and a strengthened school health are recommended.

### Neonatology

Jaundice is common in the newborn period and early discharge of healthy late preterm and full term neonates may result in an increased rate of hospital re-admission due to hyperbilirubinaemia and higher risk of kernicterus. Transcutaneous bilirubin (TcB) devices estimate serum bilirubin noninvasively and predict hyperbilirubinemia. In the past years a skin bilirubin nomogram that used the BiliCheck (BC) as transcutaneous bilirubinometer was developed and validated in a European population of healthy newborns in the first 96 hours of life. Romagnoli C et al. [14] compared the predictive ability of BC in identifying neonates not at risk of significant hyperbilirubinaemia against another device used to perform TcB, the JM-103. A total of 298 infants underwent paired-sampled bilirubin measurements with the two devices. They found 100% sensitivity for both devices after 60 hours of life, with JM-103 having less false positive results. In the first 60 hours of life there were false negative results from both devices, but, again, JM-103 gave less false positive results. Furthermore, JM-103 was less time consuming and less expensive. In conclusion, both BC and JM-103 can be safely used for TcB measurements to exclude significant hyperbilirubinemia after the 60^th^ hour of life. Nevertheless, the transcutaneous pre-discharge screening should be considered only as the first step, and a careful follow-up should be planned after discharge.

Retinopathy of prematurity (ROP) is a vasoproliferative disorder of the retina that may result in a significant loss of vision and even blindness, with posterior-ROP (P-ROP) being the most aggressive type of the disease. In the last few years, due to the increased survival rate of extremely premature neonates, the overall proportion of infants with severe ROP has considerably risen. In a prospective multicentre observational study of 421 Italian infants born with a birth weight ≤750 g and/or a gestational age (GA) ≤27 weeks Borroni C et al. [15] investigated the incidence and the relative risk factors of ROP and P-ROP. They also analyzed the occurrence of surgical treatment of ROP and evaluated the short term outcome of the disease. Erythropoietin-therapy and intraventricular haemorrhage were significantly associated with ROP, while GA ≤24 weeks and sepsis were associated with P-ROP. Eighty nine infants (34%) required laser-therapy and P-ROP was an independent factor associated with the need of surgical treatment. Absence of detachment or macular fold was reported in 251 infants with ROP as a favourable outcome, while adverse outcomes occurred in 14 patients and all of them underwent surgery and showed P-ROP. Prevention of severe ROP in very preterm infants requires a multi-specialistic team. A careful follow-up should be offered particularly to infants with P-ROP because of the significantly higher risk of surgical intervention and adverse outcome of the condition.

Foetal programming means the structural and functional changing of an organism, metabolism and function of some cells, tissues and systems, that occur even despite intrauterine limitations. Events of foetal life influence the determination of physiological patterns which may manifest as disease processes in the adulthood (Barker's hypothesis). Genetic and environmental factors (poor diet in pregnancy chronic intrauterine foetal hypoxia, the effects of xenobiotics and drugs, as well as hormonal disorders) influence the phenotype of a newborn and are involved in the intrauterine programming process. The effects of foetal programming may be passed along to the next generations via not fully understood pathways, which probably include epigenetic mechanisms. Most of the mechanisms underlying this process remain unclear and need to be elucidated. Capra L et al. [16] reviewed the literature on foetal programming, and listed the main intra- and the extrauterine environmental factors that may affect the development of the foetus. Maternal diseases like depression and anxiety, epilepsy, asthma, anaemia and metabolic disorders, in particular diabetes, are able to determine alterations in growth and foetal development. The maternal lifestyle, particularly diet, exercise and smoking during pregnancy, have an important role in the development of diseases that manifest themselves both during childhood and particularly in adulthood. Finally, if the extra-uterine environment significantly differs from the intra-uterine setting, the foetus appears more prone to develop diseases. A number of indoor and/or outdoor pollutants can contribute to an increased risk of several disorders and in some cases could be easily avoided.

### Neurology

Dyke-Davidoff-Masson Syndrome (DDMS) is a rare condition characterized by cerebral hemiatrophy, controlateral hemiplegia, facial asymmetry, developmental delay, cognitive impairment and seizures. Congenital or acquired DDMS has been frequently described and in congenital DDMS structural abnormalities of the cerebral artery vessels likely result in the development of cerebral hemiatrophy. Piro E et al. [17] described the case of a 9-months-old infant who had previously received the prenatal diagnosis of unilateral left ventriculomegaly and left cerebral hemiatrophy. Early postnatal brain magnetic resonance imaging (MRI) and contrast enhanced-MRI angiography showed reduced calibre of the left middle cerebral artery that is characteristically found in patients with DDMS. The key message is that early clinical assessment, differential diagnosis and cerebral imaging including cerebral MRI angiography allow clinicians to diagnose this rare condition also in early infancy.

The decision to encourage breastfeeding in women suffering from epilepsy or bipolar disorders who are treated with antiepileptic drugs (AEDs) should be taken after a careful evaluation of the possible side-effects on the infant caused by the indirect exposure to AEDs via breast milk. To help paediatricians in coming to a final decision, Davanzo R et al. [18] provided a revision of the current literature on the use of AEDs in the breastfeeding mother. Authors presented data on AEDs pharmacokinetic parameters (half-life, maternal plasma protein binding, milk to plasma ratio, oral bioavailability). They also discussed about the most relevant clinical parameters that should be taken into account in the assessment of the breastfeeding risk, such as the theoretical infant dose, the therapeutic dose in the neonatal period and the relative infant dose associated to AEDs. AEDs have been classified into 3 categories: 1) safe AEDs (carbamazepine, valproic acid, phenytoin, phenobarbital, primidone); 2) moderately safe AEDs (gabapentin, lamotrigine, oxcarbazepine, vigabatrin, tiagabine, pregabalin, leviracetam and topiramate); and 3) contraindicated AEDs (ethosuximide, zonisamide, clonazepam and diazepam). The conclusion was that although AEDs during breastfeeding have some contradictions, most AEDs can be considered safe, and that breastfeeding should be always encouraged unless there are evidence-based informations that contraindicate it.

### Nutrition

There is growing scientific evidences on the role of breakfast as an essential part of an healthy diet. Affinita A et al. [19] analyzed breakfast related issues based on a multidisciplinary approach including history, sociology, anthropology, medicine, psychology and pedagogy. They observed as eating habits depend on population culture and vary on time, space, as well as on our own personal choices, making breakfast the most variable meal in our diet. Recent studies showed that breakfast improves cognitive function, intuitive perception and academic performance in children, and it is important in maintaining the health and well-being of an individual not only in adulthood but also in the elderly. However, epidemiological data from industrialized countries revealed that many individuals either eat a nutritionally unhealthy breakfast or skip it completely. The authors concluded that the historical, bio-psychological and educational value of breakfast in our culture is extremely important and should be recognized and stressed by the scientific community. Efforts should be done to promote this practice for the individual health and well-being.

Acute and chronic malnutrition is frequent but often unrecognized in hospitalized children. Validated and easy methods for routine screening of nutritional status in children are lacking. Spagnuolo MI et al. [20] studied the diagnostic accuracy of the Screening Tool for Risk of Impaired Nutritional Status and Growth (STRONGkids), a 4 item score, to estimate the risk of malnutrition in children admitted to hospital. They showed that STRONGkids was easy to use. However, its usefulness in practice was limited as it had a high negative predictive value, but the positive predictive value was low. It is time to move forward and overcome the residual problem that remains with assessment of malnutrition.

## Conclusions

Also this year has been a very interesting year for pediatric research, with regard to the prevention of allergic diseases, the clinical aspects of genetic diseases, the diagnosis of neonatal pathology and therapy of autoimmune diseases and viral infections. The results of published works constitute an important support to improve the knowledge and management of diseases in childhood.

## Competing interests

The authors declare that they have no competing interests.

## Authors’ contributions

CC conceived the study, participated in its design carried out the literature research and helped to draft the manuscript. FSa participated in the design of the study, carried out the literature research and helped to draft the manuscript. AV, CPD, VM, FSp carried out the literature research and helped to draft the manuscript. SB conceived the study, and participated in its design and coordination and helped to draft the manuscript. All authors read and approved the final manuscript.
